# Study of streptomycin-induced ototoxicity: protocol for a longitudinal study

**DOI:** 10.1186/s40064-016-2429-5

**Published:** 2016-06-17

**Authors:** Adebolajo A. Adeyemo, Odunayo Oluwatosin, Olayemi O. Omotade

**Affiliations:** Institute of Child Health, College of Medicine, University of Ibadan, PMB 5017, Ibadan, Nigeria; Department of Surgery, College of Medicine, University of Ibadan, PMB 5017, Ibadan, Nigeria

**Keywords:** Ototoxicity, Streptomycin, Tuberculosis, Aminoglycosides, Africa, Drug-induced hearing loss

## Abstract

Hearing impairment is due to various causes including ototoxicity from aminoglycosides. The susceptibility to aminoglycosides increases in the presence of certain mitochondria gene mutations. There is unrestrained use of aminoglycosides in many developing nations which may worsen the burden of hearing impairment in these countries but there is lack of data to drive required policy changes. Streptomycin (an aminoglycoside) is part of the drug regimen in re-treatment of tuberculosis. Exploring the impact of streptomycin ototoxicity in tuberculosis patients provides a unique opportunity to study aminoglycoside ototoxicity within the population thus providing data that can inform policy. Also, since streptomycin ototoxicity could adversely affect treatment adherence in tuberculosis patients this study could enable better pre-treatment counseling with subsequent better treatment adherence. Patients on tuberculosis re-treatment will be recruited longitudinally from Direct Observation Therapy-Short course centers. A baseline full audiologic assessment will be done before commencement of treatment and after completion of treatment. Early detection of ototoxicity will be determined using the American Speech and Hearing Association criteria and genetic analysis to determine relevant mitochondria gene mutations will be done. The incidence of ototoxicity in the cohort will be analyzed. Both Kaplan–Meier survival curve and Cox proportional hazards tests will be utilized to determine factors associated with development of ototoxicity and to examine association between genotype status and ototoxicity. This study will provide data on the burden and associated predictors of developing aminoglycoside induced ototoxicity. This will inform public health strategies to regulate aminoglycoside usage and optimization of treatment adherence and the management of drug-induced ototoxicity among TB patients. Furthermore the study will describe mitochondrial gene mutations associated with ototoxicity in the African population.

**Electronic supplementary material:**

The online version of this article (doi:10.1186/s40064-016-2429-5) contains supplementary material, which is available to authorized users.

## Background

Hearing ability is one of the key human senses and it could be argued to be essential for satisfactory quality of life. Loss of hearing therefore, may be a major disability. This disability becomes more apparent in the communal cultural setting prevalent in many developing countries where hearing impaired people may suffer from social stigmatization and isolation. Hearing loss imposes significant social and economic disadvantages on both individuals and communities. Hearing impaired children often experience delayed development of speech, language, and cognitive skills, causing slowed learning and difficult progress in school (WHO 2014). Among adults, impairment of hearing makes it difficult to obtain and keep employment (WHO 2014). Hearing loss occurs via multiple pathways,—inflammatory disease of the middle ear is the commonest cause in children (Adeyemo 2012a)—but ototoxicity is an increasing cause of hearing loss in developing countries.

According to the World Health Organization (WHO) majority of hearing impaired people resides in developing countries; while a number of etiological factors have been identified as a cause of the impairment in these countries, ototoxicity from both antibiotics and antimalarial drugs feature prominently (WHO 2014) and aminoglycoside antibiotics are the commonest cause of ototoxicity (WHO 1994). Irrational prescription of aminoglycosides have been reported in many developing countries (Gwimile et al. 2012) and this unrestrained use may keep eliciting a huge burden of hearing impairment unless a significant policy change comes into effect in these countries. Unfortunately there is inadequate locally derived data to drive such a policy change.

Streptomycin, Amikacin, and Kanamycin are examples of aminoglycosides in the drug armamentarium against tuberculosis (Peloquin et al. 2004). Studying the streptomycin induced ototoxicity in tuberculosis patients provides an excellent opportunity to monitor aminoglycoside ototoxicity within a developing country population.

### Aminoglycoside induced ototoxicity

Ototoxicity is an irreversible side effect of aminoglycosides (De Jager and Van Altena 2002), and could manifest as either cochlea damage with permanent hearing loss or vestibular damage with dizziness, ataxia and/or nystagmus (Duggal and Sarkar 2007). This adverse effect is dose-dependent (De Jager and Van Altena 2002) and compounded by the narrow therapeutic range of aminoglycosides and the wide inter-individual variability in the pharmacokinetics of the drug (Duggal and Sarkar 2007). Aminoglycoside ototoxicity is recognized by a distinctive pattern of hearing loss, the high frequency range (4000–8000 Hz) is affected first with lower frequencies affected subsequently (Human et al. 2010).

Aminoglycosides cause degeneration of sensory cells in the cochlea; usually involving the basal turn initially before progressing to the cochlea apex. This is the basis for the initial high frequency hearing loss subsequently followed by hearing loss in the lower frequencies. Therefore in the early stages of aminoglycoside ototoxicity conversational hearing might not be affected. Vestibular impairment occurs following damage to the vestibular sensory cells in the crista ampullaris and it manifests with ataxia and nystagmus. The subject complains of gait disturbances and oscillopsia (visual blurring with head movement) in the absence of prior existence of vertigo (Kisilevsky et al. 2004). All aminoglycosides exhibit toxicity on both the cochlea and the vestibules but often not in equal proportions though tobramycin is known to affect both the cochlea and vestibules equally (Guan 2011). Aminoglycosides such as streptomycin and gentamycin are predominantly vestibulotoxic whereas neomycin and kanamycin are more toxic on the cochlea (Guan 2011).

The incidence of ototoxicity depends on the duration and dose of the aminoglycoside; reported incidence varies widely from 28 to 37 % (Sturdy et al. 2011, Peloquin et al. 2004). However factors such as definitional criteria for hearing loss, methods of assessment, subject group and treatment parameters had influenced the reported incidence of ototoxicity (Xie et al. 2011). Therefore the use of more sensitive testing criteria will probably increase the proportion of hearing loss detected in aminoglycoside exposure (Fausti et al. 1992).

### Tuberculosis control

Tuberculosis is known to man since antiquity and it is still one of the leading infectious diseases in the world with a third of the world’s population already infected (Duggal and Sarkar 2007; Jones-López et al. 2011). Nine million new cases of TB are diagnosed annually and the disease is responsible for more than two million deaths every year (Harries and Dye 2006; Adeyemo 2012b). Hopes of eradicating TB had initially brightened with the discovery of streptomycin by Waksman in 1944 (Schacht 2004) however multiple factors including resource constraints, economic crises, and recently HIV/AIDS pandemic had led to renewed surge in TB burden in many developing countries. The emergence of drug resistant TB strains are due to factors such as lack of adherence by patients to drug regimen, improper prescription, inadequate supply, and low quality drugs (Lienhardt et al. 2012). The proportion of drug resistant TB among TB cases have been increasing worldwide (Sturdy et al. 2011); since the second-line drugs advocated by WHO for the management of drug resistant TB includes aminoglycosides there has been a corresponding upsurge in the usage of aminoglycosides.

A high proportion of TB patients fail to complete their initial treatment and thus require re-treatment with streptomycin based drug regimen—category II regimen (Jones-López et al. 2011). Indeed 10–20 % of the populations with TB in developing countries are commenced on streptomycin based anti-TB drugs regimen annually (Jones-López et al. 2011) which involves daily streptomycin injection for 2 months. The key side effect of long-term administration of parenteral aminoglycosides is ototoxicity.

### Single versus multiple dosing regimen

There have been previous attempts to reduce the toxicities associated with aminoglycosides by varying the frequency of dosage. Traditionally aminoglycosides have been given in Multiple Daily Doses (MDD) in either 8 or 12 hourly doses to ensure therapeutic concentrations that could guarantee clinical success as well as preventing toxicities of the kidney or inner ear.

Single daily dosing (SDD) however might lead to greater clinical efficacy. SDD produces higher peak serum concentrations which increased the rate and proportion of the microbicidal activity (Ali and Goetz 1997). Moreover, the concept of saturation of aminoglycoside uptake into the renal proximal tubule cell also encouraged SDD practice with the intention of reducing associated nephrotoxicity (Rotstein and Mandell 2004). Additional evidence which showed that laboratory animals manifest less toxicity on SDD rather than MDD (Ali and Goetz 1997) and demonstration of saturation kinetics in the entry of aminoglycosides into the ear (Tran Ba Huy et al. 1986) probably promoted adoption of SDD.

Meta-analyses of clinical trials comparing SDD and MDD showed superiority of SDD for bacteriologic eradication and a reduced incidence of nephrotoxicity (Rotstein and Mandell 2004; Turnidge 2003). However assessment of ototoxicity in the meta-analyses did not show any clear trend as some clinical trials favored SDD while others favored MDD. No statistically significant differences were seen between SDD and MDD in the data assessment of vestibular toxicity (Rotstein and Mandell 2004). The variability in techniques of evaluating ototoxicity in the trials and lack of standard definition of what constitutes “toxicity” between the trials may had accounted for the inconsistencies in the trial results (Rotstein and Mandell 2004). The simplicity of SDD (especially in out-patient set-up), cost advantages, and efficacy have made it the preferred dosage regimen in streptomycin administration in TB control programs.

### Auditory assessment in ototoxicity

There is no universal consensus on the most appropriate way to monitor ototoxicity in patients receiving aminoglycoside therapy; the lack of consistent predictors of hearing loss is one of the shortcomings of research in human ototoxicity (Peloquin et al. 2004). In general, hearing loss due to ototoxicity is ascertained by comparing baseline data, ideally obtained before commencement of ototoxic drug administration, to the results obtained in subsequent monitoring tests (Duggal and Sarkar 2007). The duration of monitoring should extend for a reasonable period otherwise the onset of the ototoxicity may be missed as it may start at the end of the aminoglycoside therapy with slow progression (Xie et al. 2011).

Hotz et al. (1994) have proposed the use of Transiently Evoked Oto-Acoustic emission (TEOAE) for early monitoring of aminoglycoside induced ototoxicity, based on the relation of TEOAE characteristics reflecting the integrity of the cochlea’s outer hair cell (OHC). However the American Speech-Language-Hearing Association (ASHA) recommends otherwise; though the sensitivity of TEOAE to the status of OHC was acknowledged the lack of specific guidelines to tailor its application to ototoxicity monitoring was highlighted as its main Achilles’ heel (ASHA 1994). Moreover, the presence of abnormal middle-ear function and a baseline hearing loss of greater than 40 dB HL (decibels hearing level) may preclude effective monitoring of ototoxicity with oto-acoustic emissions (Harris et al. 2012).

Serial audiometry-including high frequencies—demonstrating change from baseline values in pure tone thresholds is probably the most effective method to prove ototoxic hearing loss (Duggal and Sarkar 2007). High frequency thresholds is required in the audiometry because this range (>8 kHz) is usually first (but sometimes solely) affected in ototoxicity. Limiting hearing monitoring to the conventional-frequency range may potentially result in missing many cases of ototoxicity therefore the ASHA and the American Academy of Audiology (AAA) guidelines recommends high-frequency audiometry as the method of choice in early detection of ototoxicity (ASHA 1994; AAA 2009). Additional measures should also be instituted to overcome other sources of errors in an ototoxicity screening program. Such measures will include: a thorough interview to identify other causes of conductive hearing loss from the medical history, full documentation of all drugs administered to subjects—even when such drugs are considered safe for hearing preservation—and a baseline audiogram to enable the subjects learn the test technique apart from providing a reference value for the hearing acuity (Duggal and Sarkar 2007). Since hearing loss due to aminoglycoside ototoxicity usually becomes manifest from the 5th day post commencement of drug treatment (De Jager and Van Altena 2002) post treatment screening should commence at least after a week of therapy. It has also been advocated that screening takes place at least weekly during the course of the treatment (Duggal and Sarkar 2007).

Other tests have also been advocated but critics have highlighted their shortcomings. Electro-cochleo-graphy (ECochG) requires an invasive technique to be very sensitive and a long time to acquire frequency-specific data thus eliminating its usefulness in routine hearing monitoring (ASHA 1994). Auditory brainstem (ABR) evoked response share similar limitations with ECochG in respect of time required for testing (ASHA 1994).

Despite perceived advantages of other monitoring methods audiometry remains the most widely accepted technique. The sturdiness of audiometry equipment and wide availability even in developing countries in addition to its other benefits makes it an attractive option to be adopted in field work in Africa.

### Vestibular assessment in ototoxicity

The true incidence of vestibular ototoxicity has been underestimated. Multiple factors are responsible for the lack of clear data on the true incidence of vestibular damage following aminoglycoside exposure. A main factor is that subclinical vestibular damage is hard to document, another factor is that the initial destruction of vestibular hair cells also occurs outside the normal active and passive head movement frequency range for the vestibular system.

Vigilance and prior anticipation of symptoms are the hallmarks of effective subjective evaluation of vestibular damage. Subjective evaluation of vestibular dysfunction involves recording complaints of dizziness, imbalance and oscillopsia made by the patient. Validated survey tools are also part of the armamentarium in subjective evaluation. A good example is the Dizziness Handicap Inventory (DHI) which allows subjects to evaluate on-going well-being (Jacobson and Newman 1990).

There are multiple methods for objective evaluation of vestibular function but the main goal is to determine the normal function of the labyrinthine end-organ and the Central Nervous System (CNS) pathways via the vestibulo-ocular reflex (VOR) (Kisilevsky et al. 2004). Objective evaluations of vestibular function test the integrity of the VOR; these tests include electronystagmography (ENG), rotational chair, sacculocolic testing, vestibular evoked potentials, and computerized dynamic posturography (CDP) (Kisilevsky et al. 2004). The advantages and disadvantages of each evaluation methods are shown in Table 1.

**Table 1:** Relative advantage and limitations of laboratory vestibular tests. Adapted from Kisilevsky et al. (2004)

Laboratory vestibular test	Advantages	Disadvantages
ENG caloric	Possibility of separate side stimulation Availability Highly sensitive for unilateral loss	Tests mainly lateral SCC Non-physiologic stimulus Low sensitivity in bilateral loss Tests only low frequencies
Rotating chair	Allows for high-frequency testing Physiologic stimulus Allows differentiation between central and peripheral impairment	Bilateral simultaneous stimulation only Stimulation in horizontal plane only Lower than in caloric side sensitivity
CDP	Assesses overall balance performance Directly reflects ability of everyday activity May identify abnormalities in case of normal ENG caloric Useful in rehabilitation and quantification of overall balance	Unable to localize site of lesion within vestibular system Possibility of “learning curve” in case of malingering

*CDP* computerized dynamic posturography, *ENG* electronystagmography, *SCC* semi-circular canal

ENG is the recording of eye movements -it is the most available and widely used test of vestibular function (Kisilevsky et al. 2004) and is based on the principle of measurement of changes in the corneo-retinal potential which occurs with eye movements with respect to electrodes placed around the eyes. There are multiple tests within the ENG test battery such as oculomotor test, measurement of spontaneous nystagmus, positional testing and caloric testing. The gold standard for objective evaluation of unilateral vestibular deficit is caloric test. The main advantage of the caloric test is that it is the only vestibular function test that allows separate stimulation of each labyrinth. This makes the test highly sensitive for unilateral peripheral vestibular lesions. The caloric test uses a thermal stimulus (water or air) to induce flow of the endolymph in the semi-circular canals (SCCs) through creation of a temperature gradient from one side of the canal to the other. The main canal tested is the horizontal SCC since it is the canal anatomically closet to the external auditory canal and thus it develops the largest temperature gradient (Kisilevsky et al. 2004).

The caloric test has handicaps which may restrict its use in aminoglycoside monitoring. Caloric testing is limited in bilateral vestibular loss which causes symmetrically depressed caloric responses (Fee 1980). By design caloric testing only allows for low-frequency stimulation. This is also limiting since the frequency of rotation obtainable with caloric stimulation is below the normal physiologic range of the vestibular system. This means a poor sensitivity and specificity of the caloric test for symmetric loss of vestibular function. Despite the handicaps the obvious advantages of the caloric test such as the ease of the test, the wide availability and portability of the equipment ensures that it remains a relevant tool in vestibular function studies. Combining this test with subjective evaluation techniques will enable a good monitoring system for aminoglycoside toxicity.

### Genetic mutations associated with ototoxicity

At very high cumulative drug levels most individuals will exhibit ototoxicity to aminoglycosides; while at lower cumulative drug levels, observed aminoglycoside ototoxicity is probably a result of genetic susceptibility. Mitochondria DNA abnormality was noted by Higashi (1989) to be the likely basis of the maternal inheritance observed in familial aminoglycoside ototoxicity. This observation was buttressed when Hu et al. (1991) described maternal transmission in familial aminoglycoside ototoxicity in another set of related individuals. All these observations tended to confirm that mutation of mitochondrial genes was the likely molecular basis for aminoglycoside ototoxicity (Fischel-Ghodsian 2004). Aminoglycosides exerted their antibacterial activity by direct binding to 16S ribosomal RNA (rRNA) in the 30S subunit of the bacterial ribosome, thus causing mistranslation or premature termination of protein synthesis (Chamber and Sande 1996; Noller 1991). Accumulation of non-functional and truncated proteins thus occurred leading eventually to bacterial death (Xie et al. 2011). This effect was usually limited to bacterial ribosomes because the affinity of aminoglycosides for eukaryotic ribosomes was lowered due to structural differences hence rendering the drug safe for humans (Xie et al. 2011). However since mitochondria were evolutionarily derived from bacteria, mitochondrial ribosomes therefore shared greater similarities to bacterial ribosomes than their cytosolic counterparts (Fischel-Ghodsian 2004; Guan 2004). Thus it has been suggested that aminoglycosides exerted their ototoxic activity at the mitochondrial ribosome.

The 12S rRNA ribosomal subunit binds to aminoglycosides and it is known to confer resistance to aminoglycosides in the mitochondria of bacteria and yeast (Fischel-Ghodsian 2004). Several ototoxic mitochondria DNA (mtDNA) mutations in the 12S rRNA gene have been identified in patients with aminoglycoside ototoxicity. Current reports indicated that at least five different homoplasmic mutations in the mitochondrial gene encoding 12S rRNA (*MT*-*RNR1*) have been identified to predispose individuals to irreversible aminoglycoside induced hearing loss (Bardien et al. 2009). The homoplasmic A1555G mutation and C1494T mutation and mutations at position 961 in the 12S rRNA gene have been associated with aminoglycoside-induced hearing loss (Matthijs et al. 1996; Usami et al. 1997); other identified mutations are T1095C and A827G (Thyagarajan et al. 2000; Xing et al. 2006). The A1555G mutation was the first mutation to be described and the commonest variant seen (Bardien et al. 2009); it has been reported in different populations’ worldwide (Guan 2006). The A1555G mutation leads to increased binding of aminoglycosides to the 12S rRNA ribosomal subunit with subsequent disruption of mitochondrial protein synthesis and eventual cell death (Duggal and Sarkar 2007; Qian and Guan 2009).

Identified genetic mutations appeared to be commoner in the black population. Studies in different populations have shown the A1555G mutation to be relatively rare; a frequency of 0.48, 0.09, and 0 % were seen in studies done in the general population of New Zealand, America and Argentina respectively (Scrimshaw et al. 1999; Tang et al. 2002; Gravina et al. 2007). A recent study among the Black population in South Africa however showed prevalence as high as 0.9 % (Bardien et al. 2009). This might suggests that the mutation might be more frequent among Blacks compared to other human races. Both A1555G and C1494T mutations decrease translation accuracy in the mitochondrion. This makes the ribosomal decoding site highly susceptible to damage by aminoglycosides (Bardien et al. 2009; Hobbie et al. 2008).

### Research goals

We hypothesize that the use of streptomycin based anti-TB drug regimen is associated with ototoxicity and that there is an association between genetic mutations of 12sRNA gene and development of streptomycin associated ototoxicity among West Africans.

Therefore the overall goal of this study is to determine the incidence of ototoxicity in a cohort of patients undergoing TB re-treatment and to determine any association between the genetic mutations in 12S rRNA mitochondria gene and streptomycin induced hearing loss.

Specific objectives are: (1) determine the incidence of ototoxicity in a cohort of category II TB patients; (2) determine the probability of developing ototoxicity and the median onset time of ototoxicity; (3) determine the demographic and pharmacologic parameters associated with developing ototoxicity; (4) describe the mutations in the *12S rRNA* gene in the cohort.

## Methods

### Study design

This study was approved by the Ethics Review Board of the Ministry of Health, Oyo state, Nigeria. Subjects will be recruited at the start of TB therapy and followed in a prospective longitudinal study till conclusion of treatment.

Eligible patients must meet the following criteria:

1. Diagnosis of TB and scheduled to commence TB re-treatment with streptomycin based drug regimen;
2. Hearing thresholds of 60 dB or better on pure tone audiometry (PTA) before commencement of streptomycin.

Excluded individuals will be those with:

1. Evidence of active infective pathology of the ear on examination;
2. Evidence of conductive hearing loss with an air-bone gap exceeding 10 dB on baseline PTA;
3. Asymmetric hearing exceeding 20 dB at any frequency on baseline PTA;
4. Abnormal serum creatinine at baseline;
5. Concomitant use of other ototoxic drugs (macrolides, diuretics, iron chelating drugs, chemotherapy drugs such as cisplatin and vinca alkaloids);
6. Evidence of confirmed HIV/AIDS infection.

### Study setting

Uncomplicated TB is managed in Nigeria at Directly Observed Therapy-Short course (DOTS) centers after diagnosis. These DOTs centers are located in primary health centers (PHC) within the community. PHCs are common in Nigerian towns and villages; they are sited with the goal of easy accessibility by the public. These centers are often manned by nurses, community health workers and community health extension workers. Initial diagnosis of TB (i.e. no prior treatment of TB) is designated as category I and managed with a 6 month regimen of Isoniazid, Rifampicin, Ethambutol and Pyrazinamide. However patients who default from treatment or represent with a relapse of TB are designated as category II and commenced on 8 month regimen of anti-TB medications which include daily streptomycin injection in the first 2 months. The dosage varies with weight of the patient. (See Additional file 1 for recommended streptomycin dosages by the National Tuberculosis and Leprosy Control Program of Nigeria) Study subjects will be recruited from the category II patient pool.

Subjects will be recruited consecutively as diagnosis is made and consent given at the DOTS centers. Subsequent follow-up will also take place at the DOTS centers over the course of the treatment regimen. All the relevant socio-demographic and clinical data will be recorded using an interviewer administered modified questionnaire (Katijah et al. 2009). Additional information will be retrieved from patient case notes.

### Sample size and power calculations

A recent study reported the proportion of TB patients with ototoxicity as 28 % (Sturdy et al. 2011). Assuming a 5 % level of significance, a sample size of 150 would be able to estimate a prevalence of 28 % with a precision of ±7.2 %, a prevalence of 50 % with a precision of ±8 %, and a prevalence of 5 % with a precision of ±3.5 %. The sample size will also allow the investigation of the association between ototoxicity and risk factors. For example, assuming a proportion with ototoxicity of 20 %, the study will be powered to detect an odds ratio of at least 2.5 if the prevalence of the risk factor in the population is at least 20 %. Adjustments of 10 % for loss to follow up gives a minimum sample size of 168 participants.

### Study measures at baseline and follow-up visit

Baseline pre-treatment full audiologic assessment will be performed on all the patients and repeated at completion of the TB treatment. Baseline and exit audiologic tests will include both air and bone-conduction threshold measurements in all the patients. Air conduction PTA will be done weekly during the intensive phase (2 months) and monthly during the continuation phase (6 months) till completion of the TB treatment. Enquiry on subjective hearing and balance changes will be sought from the subjects at every contact. Subjects who complain of changes will undergo repeat full audiologic evaluation. The treatment guidelines of the WHO stipulate daily intramuscular streptomycin injection for category II TB patients (WHO 2010) therefore multiple dosing regimen of streptomycin will not be considered in this study.

Baseline pure tone audiograms between 250 and 16,000 Hz will be performed for all the patients using a KUDU wave audiometer (Geoaxon, Pretoria, South Africa). This is a portable device run by an eMoyo algorithm on a laptop and it obviates the need for a sound proof booth (Swanepoel et al. 2010). The audiometer uses external audio cups to attenuate environmental noise and intra-aural probe and fore head vibrator to deliver the pure tones. It also monitors ambient sound via an inbuilt microphone and the test is stopped once the environmental sound becomes loud enough to interfere with the examination. A user friendly game pad is also attached to the laptop and user is instructed to press the keys after perceiving the pure tones. This enables the software to plot the user responses directly on an audiogram. The software also prompts the examiner to position the forehead vibrator after completion of the air conduction tests; at the end of both the bone and air conduction test the pure tone average is automatically calculated and a printer friendly audiogram is generated.

The quietest place in the DOTS center will be sought to perform the PTA. The vestibular function test (Romberg Test) will be done in an examination room with an even floor and good lighting. The patient will be put at ease and the tests to be done fully explained in a language familiar to the patient. Assurances will be given that the examiner and research assistants will catch the patient and prevent a fall in case dizziness ensues in the course of the examination (see Table 2).

**Table 2:** Measurement schedule

Measure	Time point		
	Baseline	Weekly (first 8 weeks)	Monthly (last 6 months)
Audiometry			
High frequency audiometry	•	•	•
Vestibular tests			
Questioning on vestibular symptoms	•	•	•
Romberg	•		•
	•		•
	•		•
Caloric irrigation test^a^			•
Venipuncture			
Blood sample for genetic assay	•		
Serum creatinine	•		
Patient characteristics			
Socio-demographic variables	•		
Medical history	•		

^a^Monthly assessment will be done once except clinical symptoms dictate otherwise

A panel of five homoplasmic mutations in the *12S rRNA* gene previously described to predispose individuals to ototoxicity following aminoglycoside therapy will be investigated. These are: A1555G, 961delT + C(n), T1095C, C1494T and A827G (Bardien et al. 2009). All subjects will have blood samples drawn and DNA will be extracted from whole blood samples using standard protocols. The DNA will be stored at −20 °C until batch testing is done. Genetic analysis for suspected mitochondrial polymorphisms will then be done on extracted DNA using standard protocols. Magnification of the mitochondrial DNA by PCR in 24 overlapping fragments will be done according to previously described protocol (Rieder et al. 1998). Direct sequencing of the PCR products will then be done with an ABI 3700 automated DNA sequencer; the sequences will be analyzed by comparison with the reference sequences in the revised Cambridge reference sequence (rCRS) of the human mtDNA (accession no. AC_000021. 2) (Men et al. 2011).

### Study outcomes

The criteria that will be used for identifying early ototoxic threshold shift from baseline audiogram are: (1) 20 dB or greater decrease at any one test frequency, (2) 10 dB or greater decrease at any two adjacent frequencies, or (3) loss of response at three consecutive frequencies where responses were previously obtained (ASHA 1994; AAA 2009). All the pure tone threshold shifts will be recorded at each frequency tested with reference to the baseline pure tone threshold at the same frequency. All the changes will be confirmed by retest on the same day. The degree of ototoxicity will be graded according to the National Cancer Institute’s Common Terminology Criteria for Adverse Events v4.3 (NCI 2010). Patients complaining of or showing audiological evidence of ototoxicity will be referred to the managing physicians for clinical review.

### Statistical analysis

The data will be analyzed with Stata (Ver. 12) [Stata Corp, College Station, TX, USA]. Patient baseline characteristics will be summarized with descriptive statistics and incidence rates of ototoxicity per 1000 person-years will be computed.

Person-time will be accrued from the date of recruitment into the study until the earliest of: (1) development of ototoxicity; (2) loss to follow-up (defined as missing scheduled clinic visit and not returning during the study period); (3) transfer to another facility for any reason; or (4) completion of follow-up. For all patients, time zero will be the date of recruitment into the study.

Kaplan–Meier curves and the log-rank test will be used to compare progression to ototoxicity between groups. We will use Cox proportional hazards models to determine factors associated with development of ototoxicity and also to examine for associations between the A1555G mutation in the 12sRNA gene and development of toxicity. The following variables will be measured and considered for inclusion in regression models: socio-demographic variables like age, gender, weight, education and socio-economic status; clinical variables, BMI, total cumulative dose of strep, etc. The level of significance to be used is α = 0.05.

## Discussion

This is a feasible prospective longitudinal study of streptomycin induced ototoxicity in a developing country. The protocol has not yet been fully explored before in a study setting but we believe it is robust enough to achieve the set objectives. Recruiting and following up the patients from DOTS centers will ensure that there will be minimal loss to follow up. The incidence of TB in Nigeria is 133 per 100,000 (Ibrahim et al. 2014); implying there will be a considerable population of TB patients that will progress to aminoglycoside based therapy.

Long distance to health centers have been identified as a reason for interruption of treatment of TB (Ibrahim et al. 2014) therefore this study will utilize DOTS units nested within a primary health centers for recruitment of study participants. The primary health care centers are widely distributed within communities thereby ensuring ease of access. Within these DOTS units drugs are provided free through a joint government and private donor’s initiative. The free drugs and ease of access to health care delivery point are incentives for patients to access treatment.

Despite the resurgence in incidence of TB in sub-Saharan Africa and the relatively huge numbers of people that will commence aminoglycoside based anti-TB drug regimen very little is known or being done about the ototoxicity that may arise from the aminoglycoside component of the drug regimen. It is possible that the auditory toxicities will have a negative impact on compliance with treatment. If that is true it will impair efforts to control the disease in the society. The lack of clarity in patient counseling was identified as a deterrent to treatment adherence in TB management (Ibrahim et al. 2014). Exploring the impact of aminoglycosides on the hearing and balance ability of patients will enable more accurate pre-treatment counseling sessions for patients. This will have a beneficial effect on treatment adherence. Data on genetic susceptibility will also benefit maternal relations of index participants when considering uptake of aminoglycoside based therapy.

More importantly aminoglycosides are commonly used in many developing countries. This is likely due to their cheap manufacturing costs (Xie et al. 2011) making this class of antibiotics attractive in many economically disadvantaged societies. The unrestrained use of the drugs possibly contributes to the high burden of hearing impairment in developing countries and probably a deafening of the population. Unlike in many developed societies where guidelines exists for monitoring serum concentrations when administering aminoglycosides such guidelines do not exist in many developing countries. Moreover there is lack of data to drive changes in policy regarding administration of aminoglycosides. Studying effects of streptomycin in tuberculosis patients therefore provides a unique opportunity to monitor aminoglycosides ototoxicity within the population in a developing country but the impact of the study will extend beyond tuberculosis treatment.

This study will provide insight into prevalence of ototoxicity in TB re-treatment and thus provide useful data for review of current treatment regimen and the contribution of aminoglycoside induced ototoxicity to the burden of hearing loss. It will also highlight areas requiring modification to improve treatment compliance in tuberculosis thereby providing evidence for appropriate policy formulation and resource allocation. The study will describe genetic mutations that predispose to aminoglycoside induced ototoxicity within the study cohort. There is dearth of data on these mutations in the West African population thus emphasizing further the need for this study.

## Conclusion

Hearing loss remains a chronic disability in many developing societies, reducing the burden of this disability requires a multi-dimensional strategy which will include policy shifts to tackle aminoglycoside ototoxicity.

## Additional file

10.1186/s40064-016-2429-5 Drug tables: Table of drug regimen for Tb as advocated by the National Tuberculosis and Leprosy program of Nigeria.

## Abbreviations

TB: tuberculosis
BCG: bacillus Calmette–Guérin
UNICEF: United Nations Children’s Fund
WHO: World Health Organization
MDR: multi drug-resistant
TEOAE: Transiently Evoked Oto-Acoustic emission
OHC: outer hair cell
ABR: auditory brainstem response
ASHA: American Speech-Language-Hearing Association
AAA: American Academy of Audiology
ECochG: electro-cochleo-graphy
DHI: Dizziness Handicap Inventory
CNS: Central Nervous System
VOR: vestibulo-ocular reflex
ENG: electronystagmography
CDP: computerized dynamic posturography
SCC: semi-circular canals
RNA: ribonucleic acid
DNA: deoxyribonucleic acid
PTA: pure tone audiometry
DOTS: Directly Observed Therapy-Short course
PHC: primary health centers
HFL: high frequency loss
