# Supplementary material for: Study of streptomycin-induced ototoxicity: protocol for a longitudinal study
Source: Springerplus. 2016 Jun 17;5(1):758. doi: 10.1186/s40064-016-2429-5 (PMC4912548; doi:10.1186/s40064-016-2429-5)
Supplement: Supplementary file 1 — 10.1186/s40064-016-2429-5 Drug tables: Table of drug regimen for Tb as advocated by the National Tuberculosis and Leprosy program of Nigeria. [file 40064_2016_2429_MOESM1_ESM.pdf]

Drugs tables

ADULTS

Category 1 (6 months) Regimen for New Cases: 2RHZE/4RH

Regimen	Pre-treatment weight			
	>70kg	55-70kg	38-54kg	21-37kg
Intensive phase (2 months):<br>Combined tablet of RHZE (150mg+75mg+400mg+275mg)	5	4	3	2
Continuation phase (4months):<br>Combined tablet of RH (150mg + 75mg)	5	4	3	2

Category 1 (8months) Regimen for New Cases: 2 RHZE/ 6EH

Regimen	Pre-treatment weight			
	>70kg	55-70kg	38-54kg	21-37kg
Intensive phase (2 months):<br>Combined tablet of RHZE (150mg+75mg+400mg+275mg)	5	4	3	2
Continuation phase (6months):<br>Combined tablet of EH (400mg + 150mg)	3	3	2	1

CHILDREN

Category 1 Regimen for New Cases: 2 RHZ+E/ 4EH (0-14years)

Regimen	Pre-treatment weight		
	15-20kg	8-14kg	<7kg
Intensive phase (2 months):<br>Combined tablet of RHZ (60mg+30mg+150mg)<br>Ethambutol 100mg	3<br>3	2<br>2	1<br>1
Continuation phase (4months):<br>Combined tablet of RH (60mg + 30mg)	3	2	1

The CAT 2 regimen remains 8 months: daily supervision

ADULTS

Category 2 Regimen for Relapses, Failure, RAD and Others: 2SRHZE/RHZE/5RHE

Regimen	Pre-treatment weight			
	>70kg	55-70kg	38-54kg	21-37kg
Intensive phase (3 months):<br>Combined tablet of RHZE (150mg+75mg+400mg+275mg)<br>Add in the first 2 months daily:<br>Streptomycin	5<br><br>1 gram	4<br><br>1 gram	3<br><br>0.75 gram	2<br><br>0.5 gram
Continuation phase (5months):<br>Combined tablet of RHE (150mg+75mg+275mg)	5	4	3	2

i. Streptomycin should NOT be given to pregnant women.
ii. Patients >45 years should not be given more than 0.75g of streptomycin irrespective of weight
